# Supplementary material for: Hot spot prediction in protein-protein interactions by an ensemble system
Source: BMC Syst Biol. 2018 Dec 31;12(Suppl 9):132. doi: 10.1186/s12918-018-0665-8 (PMC6311905; doi:10.1186/s12918-018-0665-8)
Supplement: Supplementary file 2 — All relevant MATLAB code. A simple Matlab implement of our predictor. (ZIP 19,742 kb) [file 12918_2018_665_MOESM2_ESM.zip › Additional file 2/Read ASA/ASA website/SKEMPI/1EMVA.htm]

 NetSurfP - Protein Surface Accessibility and Secondary Structure Predictions

  

|  |  |  |
| --- | --- | --- |
|  |  | NetSurfP - Protein Surface Accessibility and Secondary Structure Predictions  Technical University of Denmark |

  


---

```
# For publication of results, please cite:
# A generic method for assignment of reliability scores applied to solvent accessibility predictions.
# Bent Petersen, Thomas Nordahl Petersen, Pernille Andersen, Morten Nielsen and Claus Lundegaard
# BMC Structural Biology 2009, 9:51 doi:10.1186/1472-6807-9-51
#
# Column 1: Class assignment - B for buried or E for Exposed - Threshold: 25% exposure, but not based on RSA
# Column 2: Amino acid
# Column 3: Sequence name
# Column 4: Amino acid number
# Column 5: Relative Surface Accessibility - RSA
# Column 6: Absolute Surface Accessibility
# Column 7: Z-fit score for RSA prediction
# Column 8: Probability for Alpha-Helix
# Column 9: Probability for Beta-strand
# Column 10: Probability for Coil
E L  1EMVA                  1    0.664 121.597  -1.309   0.053   0.005   0.942
E K  1EMVA                  2    0.566 116.467   0.714   0.257   0.016   0.727
E H  1EMVA                  3    0.573 104.156  -0.046   0.339   0.016   0.645
E S  1EMVA                  4    0.517  60.628   1.253   0.430   0.016   0.555
B I  1EMVA                  5    0.168  30.988  -0.777   0.430   0.016   0.555
E S  1EMVA                  6    0.465  54.463  -0.249   0.339   0.016   0.645
E D  1EMVA                  7    0.508  73.203  -0.206   0.339   0.016   0.645
B Y  1EMVA                  8    0.209  44.578   0.909   0.339   0.016   0.645
E T  1EMVA                  9    0.350  48.573   0.481   0.181   0.016   0.803
B E  1EMVA                 10    0.253  44.269   0.401   0.858   0.002   0.139
E A  1EMVA                 11    0.629  69.261   0.524   0.970   0.001   0.030
E E  1EMVA                 12    0.325  56.760   1.329   0.970   0.001   0.030
B F  1EMVA                 13    0.033   6.703   0.325   0.988   0.000   0.012
B L  1EMVA                 14    0.183  33.581   1.460   0.988   0.000   0.012
E Q  1EMVA                 15    0.500  89.336   1.451   0.988   0.000   0.012
B L  1EMVA                 16    0.072  13.128   0.703   0.988   0.000   0.012
B V  1EMVA                 17    0.032   4.965   0.369   0.988   0.000   0.012
E T  1EMVA                 18    0.407  56.493   1.613   0.988   0.000   0.012
E T  1EMVA                 19    0.411  57.061   1.943   0.970   0.001   0.030
B I  1EMVA                 20    0.034   6.272   0.143   0.975   0.003   0.022
B C  1EMVA                 21    0.159  22.352   0.874   0.938   0.007   0.055
E N  1EMVA                 22    0.606  88.674   1.386   0.694   0.003   0.303
E A  1EMVA                 23    0.480  52.874  -0.498   0.246   0.004   0.750
E D  1EMVA                 24    0.697 100.438  -1.162   0.053   0.005   0.942
E T  1EMVA                 25    0.471  65.328  -0.690   0.016   0.005   0.979
E S  1EMVA                 26    0.499  58.530  -0.051   0.016   0.005   0.979
E S  1EMVA                 27    0.465  54.498   0.391   0.058   0.017   0.925
E E  1EMVA                 28    0.539  94.181  -0.080   0.782   0.003   0.216
E E  1EMVA                 29    0.688 120.194   0.955   0.923   0.002   0.076
E E  1EMVA                 30    0.252  44.024   0.991   0.923   0.002   0.076
B L  1EMVA                 31    0.067  12.323   0.265   0.970   0.001   0.030
E V  1EMVA                 32    0.391  60.051   0.462   0.970   0.001   0.030
E K  1EMVA                 33    0.538 110.687   1.849   0.970   0.001   0.030
B L  1EMVA                 34    0.032   5.859   0.644   0.970   0.001   0.030
B V  1EMVA                 35    0.058   8.899   0.942   0.970   0.001   0.030
E T  1EMVA                 36    0.487  67.575   1.455   0.970   0.001   0.030
B H  1EMVA                 37    0.221  40.273   1.453   0.970   0.001   0.030
B F  1EMVA                 38    0.025   4.997   1.081   0.923   0.002   0.076
E E  1EMVA                 39    0.371  64.761   1.355   0.923   0.002   0.076
E E  1EMVA                 40    0.468  81.829   1.650   0.938   0.007   0.055
B M  1EMVA                 41    0.050  10.085   0.967   0.802   0.014   0.185
B T  1EMVA                 42    0.135  18.766   0.465   0.561   0.047   0.393
E E  1EMVA                 43    0.524  91.473   0.228   0.191   0.086   0.723
B H  1EMVA                 44    0.169  30.668   1.053   0.058   0.017   0.925
B P  1EMVA                 45    0.237  33.602  -0.230   0.018   0.019   0.964
E S  1EMVA                 46    0.621  72.781  -0.513   0.018   0.019   0.964
E G  1EMVA                 47    0.423  33.259  -1.508   0.018   0.019   0.964
E S  1EMVA                 48    0.345  40.446  -1.246   0.018   0.019   0.964
B D  1EMVA                 49    0.238  34.310  -1.250   0.004   0.085   0.910
B L  1EMVA                 50    0.047   8.569   0.303   0.004   0.616   0.381
B I  1EMVA                 51    0.045   8.417   0.200   0.002   0.816   0.182
B Y  1EMVA                 52    0.075  16.070   0.029   0.002   0.816   0.182
B Y  1EMVA                 53    0.154  32.953   1.487   0.004   0.514   0.481
B P  1EMVA                 54    0.080  11.409   0.308   0.004   0.138   0.858
E K  1EMVA                 55    0.669 137.552   0.676   0.018   0.019   0.964
E E  1EMVA                 56    0.759 132.615   0.098   0.016   0.005   0.979
E G  1EMVA                 57    0.624  49.148  -1.513   0.016   0.005   0.979
E D  1EMVA                 58    0.619  89.227  -1.494   0.016   0.005   0.979
E D  1EMVA                 59    0.629  90.711  -0.981   0.018   0.019   0.964
E D  1EMVA                 60    0.370  53.375  -1.733   0.018   0.019   0.964
E S  1EMVA                 61    0.248  29.077   0.452   0.053   0.005   0.942
B P  1EMVA                 62    0.145  20.618  -1.112   0.502   0.002   0.495
E S  1EMVA                 63    0.461  53.994   0.602   0.694   0.003   0.303
E G  1EMVA                 64    0.246  19.400   0.325   0.858   0.002   0.139
B I  1EMVA                 65    0.032   5.920   0.531   0.970   0.001   0.030
B V  1EMVA                 66    0.067  10.298   0.489   0.975   0.003   0.022
E N  1EMVA                 67    0.500  73.259   1.490   0.970   0.001   0.030
B T  1EMVA                 68    0.151  20.888   1.652   0.970   0.001   0.030
B V  1EMVA                 69    0.031   4.780   0.986   0.970   0.001   0.030
E K  1EMVA                 70    0.367  75.430   1.498   0.970   0.001   0.030
E Q  1EMVA                 71    0.532  94.944   2.060   0.970   0.001   0.030
B W  1EMVA                 72    0.072  17.388   0.872   0.970   0.001   0.030
B R  1EMVA                 73    0.227  51.937   1.042   0.970   0.001   0.030
E A  1EMVA                 74    0.639  70.396   0.495   0.923   0.002   0.076
E A  1EMVA                 75    0.632  69.635   0.211   0.694   0.003   0.303
E N  1EMVA                 76    0.518  75.850   0.390   0.321   0.003   0.675
E G  1EMVA                 77    0.621  48.881  -1.683   0.053   0.005   0.942
E K  1EMVA                 78    0.344  70.843   0.816   0.016   0.005   0.979
E S  1EMVA                 79    0.565  66.253  -0.800   0.016   0.005   0.979
E G  1EMVA                 80    0.320  25.200  -1.496   0.018   0.019   0.964
B F  1EMVA                 81    0.175  35.183  -2.225   0.058   0.017   0.925
E K  1EMVA                 82    0.590 121.260   0.697   0.018   0.019   0.964
E Q  1EMVA                 83    0.887 158.472   0.884   0.003   0.003   0.994

Explain the output.  Go back.
```

---
